# Transplantation of Autologous Differentiated Urothelium in an Experimental Model of Composite Cystoplasty

**DOI:** 10.1016/j.eururo.2010.12.004

**Published:** 2011-03

**Authors:** Alex Turner, Ramnath Subramanian, David F.M. Thomas, Jennifer Hinley, Syed Khawar Abbas, Jens Stahlschmidt, Jennifer Southgate

**Affiliations:** a Jack Birch Unit for Molecular Carcinogenesis, Department of Biology, University of York, York, UK; b Paediatric Urology, Leeds Teaching Hospitals NHS Trust, St James's University Hospital, Leeds, UK; c Central Biomedical Services, University of Leeds, Leeds, UK; d Histopathology, Leeds Teaching Hospitals NHS Trust, St James's University Hospital, Leeds, UK; e Royal Manchester Children's Hospital, Oxford Road, Manchester, UK

**Keywords:** Bladder, Reconstruction, Urothelium, Tissue engineering, Surgical model, Enterocystoplasty

## Abstract

**Background:**

Enterocystoplasty is associated with serious complications resulting from the chronic interaction between intestinal epithelium and urine. Composite cystoplasty is proposed as a means of overcoming these complications by substituting intestinal epithelium with tissue-engineered autologous urothelium.

**Objective:**

To develop a robust surgical procedure for composite cystoplasty and to determine if outcome is improved by transplantation of a differentiated urothelium.

**Design, setting, and participants:**

Bladder augmentation with in vitro–generated autologous tissues was performed in 11 female Large-White hybrid pigs in a well-equipped biomedical centre with operating facilities. Participants were a team comprising scientists, urologists, a veterinary surgeon, and a histopathologist.

**Measurements:**

Urothelium harvested by open biopsy was expanded in culture and used to develop sheets of nondifferentiated or differentiated urothelium. The sheets were transplanted onto a vascularised, de-epithelialised, seromuscular colonic segment at the time of bladder augmentation. After removal of catheters and balloon at two weeks, voiding behaviour was monitored and animals were sacrificed at 3 months for immunohistology.

**Results and limitations:**

Eleven pigs underwent augmentation, but four were lost to complications. Voiding behaviour was normal in the remainder. At autopsy, reconstructed bladders were healthy, lined by confluent urothelium, and showed no fibrosis, mucus, calculi, or colonic regrowth. Urothelial morphology was transitional with variable columnar attributes consistent between native and augmented segments. Bladders reconstructed with differentiated cell sheets had fewer lymphocytes infiltrating the lamina propria, indicating more effective urinary barrier function.

**Conclusions:**

The study endorses the potential for composite cystoplasty by (1) successfully developing reliable techniques for transplanting urothelium onto a prepared, vascularised, smooth muscle segment and (2) creating a functional urothelium-lined augmentation to overcome the complications of conventional enterocystoplasty.

## Take Home Message

We describe successful application of composite enterocystoplasty in a porcine model comprising an augmenting smooth-muscle segment lined by in vitro–generated autologous urothelium. None of the well-documented complications of conventional enterocystoplasty were encountered in the augmented bladders.

## 1 Introduction

Although the management of severe bladder dysfunction has been transformed by enterocystoplasty, the use of intestine to increase capacity and compliance is associated with relatively common and potentially serious complications attributable to prolonged interaction of urine and the mucus-producing, absorptive, intestinal epithelium [1]. Alternatives to conventional enterocystoplasty, such as ureterocystoplasty, have proven to be of very limited application. In light of current knowledge, the only reliable means to create a urothelium-lined neobladder of adequate functional capacity is likely to entail the use of tissue engineering.

We have previously reported the development of reliable cell-culture systems for generating clinically relevant areas of normal human urothelium in vitro [2]. In addition, we have described the concept of composite enterocystoplasty, in which autologous urothelium propagated in vitro is combined with a segment of de-epithelialised bowel at the time of surgical reconstruction [3]. This offers significant advantage over other tissue-engineering strategies as it exploits the patient's vascularised smooth-muscle tissue and requires only urothelium to be engineered in vitro.

The concept of composite cystoplasty was successfully piloted in a surgical minipig model using autologous cultured urothelium combined with vascularised uterine smooth muscle [3]. While proof of principle was demonstrated, histologic analysis indicated incomplete urothelial coverage and some regrowth by residual foci of uterine epithelium. Moreover, inflammatory changes were considered attributable to inadequate integrity of the urothelial barrier, resulting in exposure of the stroma to urine. This study was designed to address these problems by:

• Promoting rapid functional integrity of the cystoplasty by using differentiated, rather than proliferative, urothelium. To this end, we have already demonstrated the potential of cultured, normal, human and porcine urothelial cells to form stratified, differentiated urothelia with barrier properties as assessed by high, transepithelial electrical resistance and low permeability [4,5].
• Confirming the feasibility of composite cystoplasty using de-epithelialised colonic segments. Technical difficulty in de-epithelialising the thin-walled porcine colon necessitated the use of uterine smooth muscle in our earlier study [3]. But, since it is envisaged that colon would be used for composite cystoplasty in humans, we modified and applied a technique of extraluminal dissection [6].
• Confirming whether urothelium lining the augmenting segments originates from cultured cells implanted at the time of reconstruction or ingrowth from surrounding native tissues. To this end, a reporter gene was integrated into cultured porcine urothelial cells for subsequent immunodetection.

## 2 Materials and methods

### 2.1 Animal procedures

Large-White hybrid (LWH) (*Sus scrofa*) female pigs weighed 25–30 kg. All procedures were undertaken in compliance with statutory regulations using standard protocols. Preliminary studies were performed using fresh bladder and colon from LWH pigs from an abattoir.

### 2.2 Harvest of urothelium

The bladder was delivered through a lower, midline, abdominal incision, opened in the midline, and a 1–2-cm^2^ area of full-thickness tissue harvested and transferred into transport medium [2] with 1 μg/ml amphotericin B, 100 U/ml penicillin, and 100 μg/ml streptomycin. The bladder and abdomen were closed with absorbable sutures.

### 2.3 Normal porcine urothelial cell culture

Normal porcine urothelial (NPU) cells were isolated and propagated as described [3]. Briefly, bladder tissue was incubated in 0.5% (weight per volume) dispase II (Roche Diagnostics Ltd, West Sussex, UK) to separate the urothelium, which was collected, treated with collagenase, and seeded into Primaria culture flasks (BD Falcon, Becton, Dickinson UK, Oxford, Oxfordshire, UK) at 4 × 10^4^ cells per square centimetre in antibiotic-free, keratinocyte serum-free medium (KSFM) containing recombinant epidermal growth factor and bovine pituitary extract (Invitrogen Ltd, Paisley, UK) and 30 ng/ml cholera toxin (Sigma-Aldrich, St. Louis, USA) as KSFM complete (KSFMc) [2]. Cultures were maintained at 37 °C and subcultured at just confluence [2].

#### 2.3.1 Stable integration of enhanced green fluorescent protein (eGFP) into porcine urothelial cells

The plasma eGFP-C1 (pLeGFP-C1) retroviral vector was transfected into PT67 packaging cells as recommended (Clontech, Mountain View, CA, USA). Replication-deficient virions were collected and used to transduce NPU cells, which were selected and maintained in KSFMc containing 0.1 mg/ml G418 sulphate. Assessment of eGFP expression was performed by direct epifluorescent microscopy or, following formation and harvest of urothelial cell sheets (see below), formalin-fixed, paraffin wax-embedded sections were immunoperoxidase labelled using a monoclonal antibody against GFP (Table 1 and Section 2.3.4).

#### 2.3.2 Differentiation and cell sheet formation

NPU cells were switched from proliferative to differentiated phenotype as described [5]. NPU cell cultures were subcultured in medium containing 5% bovine serum; cell sheets were formed 16 h prior to surgery by adjusting the calcium concentration to 2.5 mM before harvest using dispase [3]. In preliminary experiments, the viability of urothelial cell sheets was tested by transfer onto a de-epithelialised stroma and maintenance in organotypic culture [7].

**Fig. 1:**
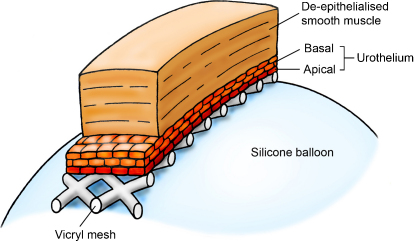
Maintenance of urothelial polarity by association with Vicryl mesh to form the delivery vehicle for transplant onto the seromuscular segment during composite cystoplasty. The Vicryl covers the luminal aspect of the reconstruction, acting as a protective layer apposed to the intravesical balloon and providing a substrate for suture.

For surgical transplantation, a trimmed Vicryl mesh from a renal prosthesis bag (VM102, Ethicon, Berkshire, UK) was placed onto the surface of the detaching cell sheet to facilitate transfer to the de-epithelialised bowel while maintaining correct urothelial polarity (Fig. 1).

#### 2.3.3 Composite cystoplasty

**Fig. 2:**
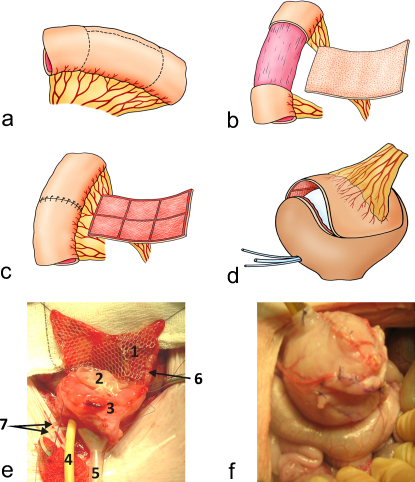
The different stages of composite cystoplasty. (a) To develop a vascularised, de-epithelialised seromuscular segement, a Foley catheter was inserted rectally and the balloon filled with sterile water within the portion of sigmoid bowel to be dissected. The dissection limits of the bowel and mesentery were defined; (b) Using the balloon as a support, the seromuscular layer of the bowel was incised and separated from the lamina propria deep to the mucosa; (c) The vascularised, deepithelialised patch was isolated and received the autologous tissue engineered urothelial cell sheet in complex with the Vicryl mesh. The mesenteric fenestration was closed to prevent internal hernia; (d) The bladder was opened widely and augmented with the composite bowel segment. Gentle distension of the augmented bladder was maintained with a silicone vesical conformer. Urine was diverted postoperatively with ureteric stents and a Malecot suprapubic catheter. Stages (c) and (d), respectively, are illustrated in (e) and (f) during an actual composite cystoplasty operation. (e1) Patches of Vicryl mesh supporting urothelial cell sheets against de-epithelialised colon; (e2)vesical conformer (collapsed); (e3) opened native bladder; (e4) Malecot suprapubic catheter; (e5) filling tube for vesical conformer; (e6) detubularised colon; (e7) ureteric catheters.

The technique of extramucosal colonic dissection was used to develop a vascularised, de-epithelialised, seromuscular segment of mean size 17.5 cm^2^ (range: 15–21 cm^2^). A Foley catheter was inserted rectally and the balloon filled with sterile water within the portion of sigmoid bowel to be dissected (Fig. 2a). The dissection limits of the bowel and mesentery were defined and mesenteric fenestrations were made. Using the balloon as a support, the seromuscular layer of the bowel was incised and separated from the lamina propria deep to the mucosa (Fig. 2b). The vascularised, de-epithelialised patch was isolated and received the autologous tissue-engineered urothelial sheet in complex with the Vicryl mesh, which was secured in place using 8/0 Vicryl sutures and maintaining the normal polarity of the urothelium (Fig. 2c). The seromuscular edges from the remaining bowel were apposed and sutured, “intussuscepting” the devascularised mucosa. The mesenteric fenestration was also closed to prevent internal hernia. The bladder was opened widely and augmented with the composite bowel segment (Fig. 2d). Gentle distension of the augmented bladder was maintained with a silicone vesical conformer (Silimed, Rio de Janeiro, Brazil), which served to gently appose the graft to the de-epithelialised stroma. Urine was diverted postoperatively with ureteric stents (Vygon 4FG feeding tubes; Vygon, Ecouen, France) and a Malecot suprapubic catheter (Rusch; Teleflex, Limerick, PA, USA). The abdomen was closed; catheters, drains, and tubing were secured externally with sutures.

Catheters and the deflated conformer were removed under sedation after 2 wk. Postsacrifice at 3 mo, bladders were collected and processed for immunohistology.

#### 2.3.4 Immunohistology

Native and augmented bladder segments were trimmed and fixed in 10% formalin for 24 h. Tissue was dehydrated through ethanol into xylene and embedded in paraffin wax. Dewaxed five micrometre sections were either stained with haematoxylin and eosin to define the overall structure of the augmented neobladders or labelled with antibodies against specific markers. CK7, CK13, and CK18 were used to define the phenotype of the tissue in relation to the epithelial lining. UPK3a was included as a marker of urothelial differentiation. The normality of the tissues was further appraised by mitotic index, inflammation, and squamous metaplasia assessed using Ki-67, CD3, and CK14, respectively. For immunolabelling, endogenous avidin-binding sites were blocked and binding of primary antibodies was detected using an indirect streptavidin avidin-biotin immunoperoxidase technique. Antigens masked by tissue processing were retrieved either by boiling in citric acid buffer or by combining with trypsinisation (Table 1).

## 3 Results

### 3.1 Validation of cell-culture techniques

**Fig. 3:**
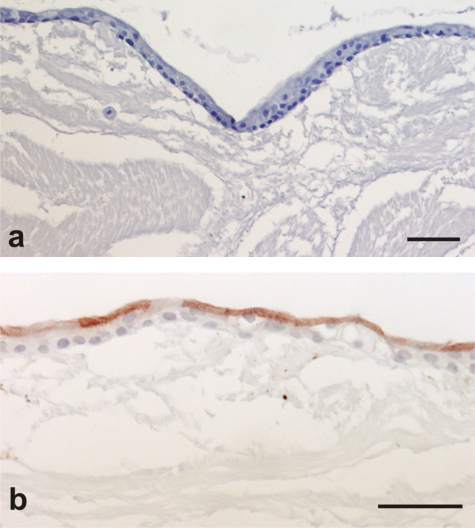
(a) Haematoxylin and eosin–stained section of stratified urothelium that showed (b) apical expression of the terminal differentiation marker, UPK3a. Scale bar: 50 μm.

The capacity for in vitro-generated, differentiated urothelial sheets to attach and survive on a de-epithelialised stroma was confirmed by organ culture (Fig. 3).

**Fig. 4:**
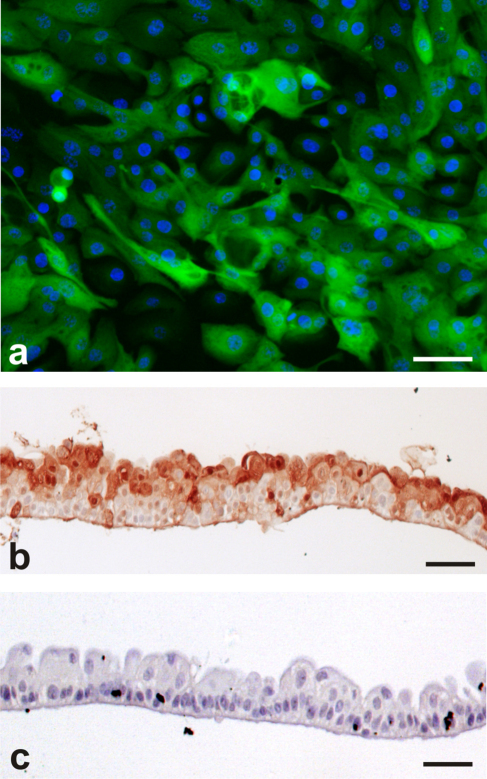
Retroviral transduction of pig urothelial cell cultures with enhanced green fluorescent protein (eGFP) as a marker protein. (a) Expression of eGFP demonstrated by epifluorescent microscopy. Nuclei are stained with Hoechst 33258 (blue). Scale bar: 10 μm. (b) Immunohistology with anti-GFP monoclonal antibody on differentiated, pig urothelial cell sheets developed from transduced pig urothelial cell culture and (c) nontransduced, pig urothelial, control cell culture to show specificity of eGFP labelling. Scale bars: 50 μm.

NPU cultures were transduced with GFP retrovirus with ≤100% efficiency. Transduced cultures were fluorescent (Fig. 4a) and there was no attenuation of eGFP expression over serial passage, despite withdrawal of antibiotic selection. Transduced cells appeared morphologically normal with no reduction in culture lifespan. eGFP could also be detected immunohistochemically on paraffin wax-processed sections of in vitro–differentiated transduced NPU sheets (Fig. 4b).

### 3.2 Extraluminal seromuscular dissection of the colon

Extraluminal dissection of the seromuscular plane was piloted in cadaveric pigs and confirmed clean separation of mucosa from the underlying musculature of the seromuscular segment. Histologically, the de-epithelialised segment appeared well-vascularised with no evidence of residual crypts. The mucosa showed complete separation from the underlying musculature with no ingress into the crypts.

### 3.3 Composite cystoplasty

#### 3.3.1 Stage 1: Bladder tissue harvest for cultivation

Bladder specimens of 1.5–2 cm^2^ were harvested from 15 animals. Successful NPU cultures were established from 13 animals, with the number of cells recovered at biopsy ranging from 0.86 × 10^6^ to 7.06 × 10^6^. These were subcultured between three and eight times to achieve clinically useful quantities ranging between 17.3 × 10^6^ and 123.40 × 10^6^ cells (mean: 55.4 × 10^6^ cells), with 10% of cells cryopreserved following the first passage. One animal was euthanised because of a large incisional hernia and another succumbed to anaesthetic complications, leaving 11 animals for reconstruction.

#### 3.3.2 Stage 2: Bladder augmentation

Bladder augmentation was performed a minimum of 4 wk after harvest of tissue for NPU culture. No technical intraoperative problems were encountered, but one animal died from an adverse reaction to sedation during catheter removal. Early in the study, three pigs died postoperatively due to sepsis secondary to urine extravasation (around a catheter in one animal and from the suture line in two animals). Thereafter precautions were taken to safeguard vascularity at the margins of the seromuscular segment and prevent leakage from the bladder at the exit site of the suprapubic catheter. Seven animals made an uncomplicated recovery and survived without complication to sacrifice at 3 mo. Normal voiding was observed to be rapidly reestablished following catheter removal and was maintained to sacrifice without complication.

#### 3.3.3 Stage 3: Analysis of reconstructed bladders

##### 3.3.3.1 Macroscopic appearance

At autopsy, all seven reconstructed bladders appeared healthy and well healed with no ulcers, mucus, calculi, or any other focal lesion within the lumen. None of the composite segments were contracted and the vascular pedicles were normal. The Vicryl mesh had completely degraded in all animals. After fixation, the graft surface area was calculated and compared with measurements obtained at the time of composite cystoplasty (Table 2). Although some augments were reduced in size, no morphologic evidence of fibrosis was found. Shrinkage of visceral tissue is known to occur not only upon fixation in formalin but also immediately after evisceration in air [15]. All bowel anastomoses were well healed and the luminal surface at the resection site appeared normal with no residual intussuscepted tissue.

##### 3.3.3.2 Histology

**Fig. 5:**
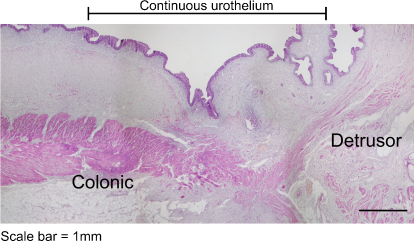
Low-power histology of augmented bladder across the anastomosis between native and augmented segments. Note the junction between colonic and detrusor smooth muscle and the uninterrupted coverage by urothelium. Scale bar: 1 mm.

A clear demarcation was present between bladder and bowel smooth muscle at the site of anastamosis (Fig. 5).

**Fig. 6:**
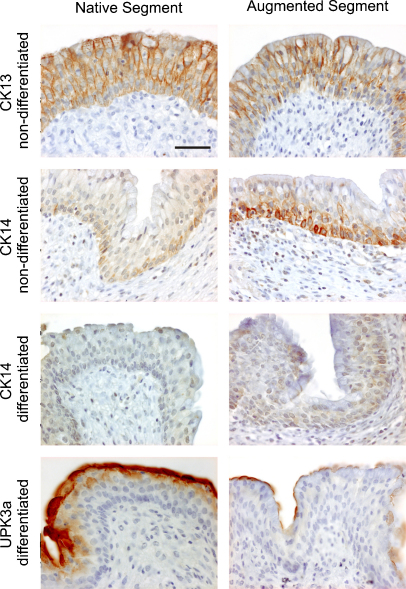
Immunohistochemistry of native and augmented bladder segments constructed using either nondifferentiated or differentiated urothelial cell sheets. Urothelium from all reconstructed bladders expressed CK13 and UPK3a, and only bladders reconstructed with nondifferentiated urothelium showed any evidence of expression of CK14. Scale bar: 50 μm.

Augmented segments were completely covered with confluent urothelium. Whereas some samples appeared purely transitional in nature, others were transitional with some columnar attributes, such as tall, aligned cells with basally located nuclei (Fig. 6, Table 3). The histology was identical in augmented and adjacent native bladder at the time of sacrifice with no differences relating to the use of nondifferentiated or differentiated urothelium at the time of augmentation. No evidence of colonic-crypt regrowth was seen in any of the augmented bladders. Inflammation was minimal, although the density of lymphocytic infiltrate in the lamina propria in bladders reconstructed with nondifferentiated urothelium was greater than with differentiated urothelium.

##### 3.3.3.3 Immunohistochemistry

**Fig. 7:**
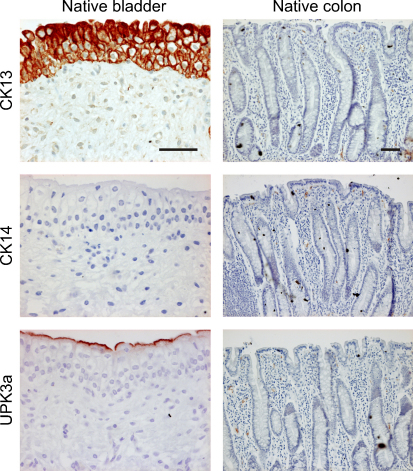
Immunohistochemistry of native pig bladder and colon. The normal expression of CK13, CK14, and UPK3a is shown for comparison with Figure 6. Note expression of CK13 and UPK3a provides markers that differentiate urothelial from colonocyte differentiation, whereas CK14 is not normally expressed by either tissue. Scale bar: 50 μm.

Expression of CK7, CK13 and CK18 was detected in native urothelium, but not colonic tissues (Fig. 7). These same isotypes were detected in the epithelium overlying the augmented segments, with the exception of CK7 in one augment constructed with nondifferentiated urothelium and CK18 in two augments constructed with differentiated urothelia. CK14 was neither expressed in native porcine urothelia nor colonic epithelia, but was expressed basally and most abundantly in the native and augmented segments of bladders constructed with nondifferentiated urothelia. UPK3a was expressed along the superficial edge of native urothelium in all augmented segments, with the exception of the bladder constructed with differentiated eGFP-expressing urothelium. Cell-cycle activity, assessed by Ki67 expression, was similar in augmented and adjacent native urothelium.

In the pig constructed with differentiated eGFP-tagged cells, no eGFP immunolabelling could be detected in the augmented (or native) segment of the bladder, although the corresponding NPU cells had expressed GFP prior to transplantation. Notably, this was the only sample that did not express UPK3a. In all other respects, the bladder constructed with eGFP-transduced cells was similar to the other reconstructed bladders.

## 4 Discussion

We have demonstrated successful implementation of composite cystoplasty for bladder reconstruction in a porcine model. Normal voiding was reestablished soon after surgery and examination of the reconstructed bladders after 3 mo demonstrated viable, vascularised, composite cystoplasty segments with no fibrosis or contraction. Augmented segments were completely lined by confluent urothelium with minimal inflammatory changes, indicating effective barrier function. In humans, UPK3a provides an objective marker of late/terminal urothelial differentiation whose expression parallels the development of a functional barrier; this relationship is more tenuous in pig urothelium, however, where UPK3a is expressed by stratified cultures that have not developed barrier properties [5]. This highlights that while the pig is a valuable model for developing and testing surgical procedures, it is not a direct substitute for human studies. In culture, NPU cells were more fastidious than normal human urothelial (NHU) cells and, despite optimising procedures for growing, differentiating, and transferring sheets of porcine urothelium, cell-culture results were inferior to equivalent results using NHU cells.

We postulated that implanting sheets of differentiated urothelium would establish more rapid, effective barrier function and noted reduced urothelial CK14 expression and fewer lymphocytes in the subaugment stroma of bladders reconstructed with differentiated urothelium. These findings support reconstruction with differentiated, rather than proliferative, urothelium.

An important aim was to overcome problems encountered previously in dissecting the thin-walled porcine colon [3]. By employing a modified technique of extraluminal dissection [6], it was possible to create well-vascularised seromuscular segments that retained viability when combined with autologous urothelium. At the time of sacrifice there was no evidence of contraction of the augmenting segments and histology revealed healthy smooth muscle with no evidence of fibrosis. Importantly, a clean plane of separation was achieved at the level of the lamina propria, thus preventing any colonic mucosal-cell regeneration from the basal crypt compartment. Compared to the pig, human sigmoid colon has a thick muscle wall and the feasibility of demucosalisation by open dissection is established [8,9]. Nevertheless, the use of extramucosal dissection has the benefit of reducing risk of sepsis by preventing peritoneal soiling with intestinal content.

Composite cystoplasty has the advantage of retaining the compliant properties imparted by the native smooth muscle of the bowel wall while limiting the requirement for tissue engineering to a single, autologous cell type (urothelium). This is a much less challenging approach than that taken by others in attempting to create a fully tissue-engineered bladder wall [10]. Whereas the properties of cultured urothelium are well documented, the biologic and functional properties of cultured smooth-muscle cells are far less well understood. Experience with composite cystoplasty is limited to a porcine experimental model that awaits objective assessment of functional outcome. However, our findings indicate that an approach which combines tissue-engineered urothelium with host smooth muscle may provide a more robust approach than one dependent upon the uncertain functionality of tissue-engineered smooth muscle.

Reported observations in experimental animals and in patients with neobladder augments derived from cultured urothelial and detrusor cells have indicated an advantage from regular postoperative distension (“bladder cycling”) to facilitate the development of adequate bladder capacity and compliance [11]. We found some evidence of a glandular type of reaction in both augmented and native segments of some reconstructed bladders. This was unrelated to the use of proliferative or differentiated urothelium and is likely to reflect an individual, nonspecific response to the presence of the catheters and balloon [12].

We have shown in organ culture that urothelial sheets form integrated basement membrane attachments to the stroma within 24 h of transplant [13], suggesting that future refinement of composite cystoplasty might include earlier removal of balloons and catheters to limit any metaplastic response to the materials.

Although it was an original aim of our study, we were unable to demonstrate unequivocally whether the urothelium of the augmented segment derived from the transplanted sheet or from ingrowth of the native urothelium. The former is most likely as the outcome reflected the type of urothelium transplanted and previous control reconstructions performed with de-epithelialised stroma did not demonstrate urothelial overgrowth [3]. However, the inability to demonstrate GFP expression in vivo meant that we were unable to address this categorically and a small possibility must remain that the transplanted urothelium provided a “feeder” effect that promoted ingrowth of native urothelium.

Finally, it is important to acknowledge that in common with virtually all experimental bladder-tissue engineering studies, this work was undertaken using animals with normal bladders. Before this technique is translated into clinical practice it will be important to evaluate the growth and differentiation capacity of urothelial cells harvested from diseased human bladders.

## 5 Conclusions

We have described successful application of composite enterocystoplasty in a porcine model, comprising an augmenting smooth-muscle segment lined by in vitro–generated autologous urothelium. None of the well-documented complications of conventional enterocystoplasty were encountered in the augmented bladders.

We suggest the approach is close to translation to human subjects once reliance on bovine factors is removed and a more efficient method to harvest urothelial cell sheets is identified, with thermoresponsive polymer technology providing one option [14].

  ***Author contributions:*** Jennifer Southgate had full access to all the data in the study and takes responsibility for the integrity of the data and the accuracy of the data analysis.  

*Study concept and design:* Thomas, Southgate.

*Acquisition of data:* Turner, Subramaniam, Abbas, Hinley.

*Analysis and interpretation of data:* Turner, Stahlschmidt, Hinley, Southgate.

*Drafting of the manuscript:* Turner, Thomas, Southgate.

*Critical revision of the manuscript for important intellectual content:* Thomas, Southgate, Turner, Stahlschmidt, Subramaniam, Abbas.

*Statistical analysis:* None.

*Obtaining funding:* Turner, Thomas, Southgate.

*Administrative, technical, or material support:* Hinley.

*Supervision:* Thomas, Southgate.

*Other* (specify): None.  

***Financial disclosures:*** I certify that all conflicts of interest, including specific financial interests and relationships and affiliations relevant to the subject matter or materials discussed in the manuscript (eg, employment/ affiliation, grants or funding, consultancies, honoraria, stock ownership or options, expert testimony, royalties, or patents filed, received, or pending), are the following: None.  

***Funding/Support and role of the sponsor:*** The Wellcome Trust supported the study as a Clinical Research Training Fellowship awarded to A. Turner. J. Southgate and J. Hinley are supported by York Against Cancer.

## Figures and Tables

**Table 1:** Antibodies used for determination of tissue phenotype

Antigen	Antibody	Source	Dilution	Antigen retrieval method*
CK7	OV TL 12/30	Novocastra	1 in 400	A
CK13	CK13.1	ICN	1 in 10	A
CK14	LL002	Serotec	1 in 500	B
CK18	CY90	Sigma	1 in 5000	A
CD3	SP7	Abcam	Undiluted	A
UP3Ka	AU1	Progen	1 in 40	A
Ki-67	MM1	Novocastra	1 in 100	A
Anti-GFP	–	Roche	1 in 400	A

* Antigens masked by tissue processing were retrieved either by boiling in citric acid buffer (method A) or combined with trypsinisation (method B), as recommended by the antibody manufacturer.

**Table 2:** Comparison of size of graft at the time of composite cystoplasty and after 3 mo

Pig	Surface area of graft. cm^2^		Change in size, %
	At composite cystoplasty	Postmortem after fixation with formalin*	
1	16	6	−62.5
2	16.25	15	−7.7
3	16.25	18	+10.8
4	18.75	22.5	+20
5	21	19.25	−8.3
6	15	22.75	+51.7
7	21	24.5	+16.7

* Nonabsorbable sutures (prolene) were placed at each corner of the posteriorly applied graft to allow accurate measurement of graft size in the last four animals. In the first three animals, margins were approximated from the macroscopic appearance of the graft.

**Table 3:** Morphologic and phenotypic properties of reconstructed bladders

Pig	Augmenting urothelium	Passages, No.	Morphology of augmented segment	Phenotype
1	Differentiated	8	Transitional with columnar attributes	Transitional; UPIIIa positive
2	Proliferative	5	Transitional	Transitional; UPIIIa positive
3	Differentiated	5	Transitional	Transitional; UPIIIa positive
4	Differentiated	7	Transitional with columnar attributes	Transitional; UPIIIa positive
5	Proliferative	3	Transitional with columnar attributes	Transitional; UPIIIa positive
6	Proliferative	4	Transitional	Transitional; UPIIIa positive
7	Differentiated plus eGFP	5	Transitional with columnar attributes	Transitional; UPIIIa negative; eGFP negative

## References

[1] Kropp B.P., Cheng E.Y., Docimo S.G., Canning D.A., Khoury A.E. (2007). Bladder augmentation: current and future techniques. Clinical Pediatric Urology.

[2] Southgate J., Hutton K.A., Thomas D.F., Trejdosiewicz L.K. (1994). Normal human urothelial cells in vitro: proliferation and induction of stratification. Lab Invest.

[3] Fraser M., Thomas D.F., Pitt E., Harnden P., Trejdosiewicz L.K., Southgate J. (2004). A surgical model of composite cystoplasty with cultured urothelial cells: a controlled study of gross outcome and urothelial phenotype. BJU Int.

[4] Cross W.R., Eardley I., Leese H.J., Southgate J. (2005). A biomimetic tissue from cultured normal human urothelial cells: analysis of physiological function. Am J Physiol Renal Physiol.

[5] Turner A.M., Subramaniam R., Thomas D.F.M., Southgate J. (2008). Generation of a functional, differentiated porcine urothelial tissue in vitro. Eur Urol.

[6] Hafez A.T., Bagli D.J., Bahoric A. (2003). Aerosol transfer of bladder urothelial and smooth muscle cells onto demucosalized colonic segments: a pilot study. J Urol.

[7] Scriven S.D., Trejdosiewicz L.K., Thomas D.F.M., Southgate J. (2001). Urothelial cell transplantation using biodegradable synthetic scaffolds. J Mat Sci Mat Med.

[8] Coran A.G., Teitelbaum D.H. (2000). Recent advances in the management of Hirschsprung's disease. Am J Surg.

[9] Jednak R., Schimke C.M., Barroso U.J., Barthold J.S., Gonzalez R. (2000). Further experience with seromuscular colocystoplasty lined with urothelium. J Urol.

[10] Atala A., Bauer S.B., Soker S., Yoo J.J., Retik A.B. (2006). Tissue-engineered autologous bladders for patients needing cystoplasty. Lancet.

[11] McLorie G.A. (2009). Editorial comment: determinants of success and failure of seromuscular colocystoplasty lined with urothelium. J Urol.

[12] Drolet R., Lee S.A., Straw B.E., Zimmerman J.Z., D’Allaire S., Taylor D.J. (2006). Diseases of the urinary system. Diseases of the Swine.

[13] Scriven S.D., Booth C., Thomas D.F., Trejdosiewicz L.K., Southgate J. (1997). Reconstitution of human urothelium from monolayer cultures. J Urol.

[14] Takahashi H., Nakayama M., Yamato M., Okano T. (2010). Controlled chain length and graft density of thermoresponsive polymer brushes for optimizing cell sheet harvest. Biomacromolecules.

[15] Goldstein N.S., Soman A., Sacksner J. (1999). Disparate surgical margin lengths of colorectal resection specimens between in vivo and in vitro measurements. The effects of surgical resection and formalin fixation on organ shrinkage. Am J Clin Pathol.

